# Supplementary material for: Mapping the evolution of fertility support policies in China: A content and instrumental analysis
Source: PLoS One. 2025 Oct 9;20(10):e0332137. doi: 10.1371/journal.pone.0332137 (PMC12510515; doi:10.1371/journal.pone.0332137)
Supplement: S1 Appendix — (ZIP) [file pone.0332137.s001.zip › S1 Appendix. 226 original policy documents/88-交通部关于发布《交通女职工劳动保护实施办法》的通知(FBM-CLI-4-28228).doc]

交通部关于发布《交通女职工劳动保护实施办法》的通知

**发布部门：** [交通部(已撤销)](https://www.pkulaw.com/law/chl?Aggs.IssueDepartment=60216) [机构沿革](javascript:void(0))

**发文字号：**交人劳发〔1995〕419号

**发布日期：**1995.05.12

**实施日期：**1995.07.01

**时效性：** [失效](https://www.pkulaw.com/law/chl?Aggs.TimelinessDic=02)

**效力级别：** [部门规章](https://www.pkulaw.com/law/chl?Aggs.EffectivenessDic=XE0301)

**法规类别：** [劳动安全与劳动保护](https://www.pkulaw.com/law/chl?Aggs.Category=08706)

**失效依据：**[交通运输部关于废止37件交通运输规章的决定](javascript:TMP(240643,0,'chl'))

　　　　　 交通部关于发布《交通女职工劳动保护实施办法》的通知
　　　　 （交人劳发（１９９５）４１９号　１９９５年５月１２日）

各省市、自治区交通厅（局）、部直属及双重领导单位和工会：
　　为贯彻落实国务院１９８８年７月２１日发布的《[女职工劳动保护规定](https://www.pkulaw.com/chl/133408887a349cdabdfb.html)》，结合交通系统的行业特点与实际情况，特制定《交通女职工劳动保护实施办法》，现予印发，请认真贯彻执行。

附：　　　　　　　 交通女职工劳动保护实施办法

**第一条**　为了切实搞好女职工劳动保护，根据国务院颁布的《[女职工劳动保护规定](https://www.pkulaw.com/chl/133408887a349cdabdfb.html)》，结合交通系统实际情况，制定本办法。

　　**第二条**　本办法适用于交通系统各企业、行政、事业单位的女职工。

　　**第三条**　各单位必须严格执行国家有关女职工劳动保护的法规和交通部的有关规定，并确定负责女职工劳动保护的工作机构或专兼职人员，加强管理和监督。

　　**第四条**　凡适合妇女从事劳动（国家规定的不适合妇女的工种或者岗位除外）的单位，不得以性别为由拒绝录用女职工，或者提高录用标准。

　　**第五条**　在女职工怀孕期、产期、哺乳期工资按国家规定发放，不得影响工资调整及有关福利待遇，不得因此停薪或提前解除劳动合同（如合同期已满，必须延续到孕期、产假和哺乳期满），不得以女职工上述生理特点为由，将其转为待聘、编余人员或辞退。

　　**第六条**　禁止安排女职工从事国家规定的第四级体力劳动强度的劳动以及其他女职工禁忌从事的劳动。

　　**第七条**　月经期保健
　　（一）宣传普及月经卫生知识。
　　（二）在同一工作场所、女职工在１００人以上有条件的单位，应逐步建立女职工卫生室，健全相应的制度。女职工在１００人以下的有条件的单位，应逐步设置简易的温水箱、消毒箱及冲洗器。暂时不具备条件的单位，要积极创造条件，建立女职工卫生室或采取相应的措施。对流动、分散、野外工作的女职工应发放单人自用冲洗器。
　　（三）从事下列劳动的女职工，在月经期内暂时调整工作或给予１至２天的休假，并按出勤计算；
　　１、国家规定的第三级体力劳动强度的劳动；
　　２、《高处作业级》标准中第二级（含二级）以上的作业；
　　３、食品冷冻库及冷水、低温（低于五摄氏度）等作业；
　　４、野外流动作业、长久站立、行走、蹲位作业。
　　（四）患有重度痛经及月经量过多者经合同医院或妇幼保健机构确诊证明，月经期间可适当给予１至２天的休假。
　　（五）女职工月经卫生费每月不低于４元钱。

　　**第八条**　孕期保健：
　　（一）对已孕女职工建立孕产妇系统保健卡，建卡率要求达９５％以上，怀孕３个月开始填写保健卡，按卫生部门的要求定期进行产前检查，检查率要求达９０％以上。对高危孕妇，所在单位应配合医院严密观察和监护，使高危孕妇管理率达到１００％。
　　（二）对妊娠的女职工，所在单位不得安排其从事国家规定的第三级体力劳动强度的劳动和孕妇禁忌从事的劳动，不得在正常劳动日以外延长劳动时间；对不能适应原劳动的，应根据合同医院证明，予以减轻劳动量或安排其他劳动。从事野外勘测工作及施工作业、公路养护、高等级公路收费、汽车司乘、港口装卸作业、轮船餐服、施工班组工作的女职工怀孕满５个月，应安排其他适当工作。
　　（三）女职工较多的单位应建立孕妇休息室。对妊娠满７个月以上（含７个月）的女职工，应在劳动时间内安排１至２小时工间休息或适当减轻工作，并不得安排从事夜班劳动。
　　（四）怀孕的女职工，在劳动时间内进行产前检查，应当算做劳动时间。对生产一线的女职工，要相应地减少生产定额，以保证产前检查时间。
　　（五）孕妇孕期及分娩时在合同医院发生的检查费、接生费、手术费、住院费、药费由原医疗经费渠道开支或按《企业职工生育保险试行办法》执行。

　　**第九条**　产期保健
　　（一）女职工产假（怀孕７个月以上）为９０天，其中产前１５天，难产增加１５天。符合计划生育政策的多胞胎生育，每多生育一个婴儿，增加产假１５天，当地政府有规定并优于本办法的，也可执行当地政府的规定。教师正值寒、暑假期间生育的，可按地方政府计划生育有关规定执行。女职工在产假期间，不影响其原有福利待遇。
　　（二）女职工怀孕不满４个月流产的，应根据医院证明，给予产假１５天至３０天；怀孕４个月以上７个月以下流产时，给予产假４２天，怀孕７个月以上的按正常产休假，以上产假期间工资照发。对有过两次以上自然流产史，现又无子女的女职工，应调离有可能直接或间接导致流产的作业岗位。

　　**第十条**　哺乳期保健
　　（一）有不满１周岁婴儿的女职工，需亲自哺乳的，其所在单位应当在每班劳动时间内给予其两次哺乳（含人工喂养）时间，每次３０分钟。女职工每班劳动时间内的两次哺乳时间，可以合并使用。哺乳时间和在本单位内哺乳往返途中的时间，算做劳动时间。
　　哺乳期满时，正值炎热季节（七至八月份），可根据当地气候条件延长１至２个月。对双胞胎或婴儿虽满周岁，因疾病、体弱经医务部门证明，哺乳时间可以适当延长，但最多不超过６个月。
　　（二）女职工在哺乳期内，所在单位不得安排其从事国家规定的第三级体力劳动强度的劳动和哺乳期禁忌从事的劳动，不得延长其劳动时间及从事夜班劳动。有条件的单位，应建立哺乳室。室内有洗手设施，乳母不得穿工作服进入哺乳室。

　　**第十一条**　产后保健
　　（一）女职工产假期满，恢复工作时，允许有１至２周时间逐渐恢复原定额工作量。
　　（二）女职工产假期满后是否办理离岗休假，根据工作情况和本人自愿的原则，依照当地计划生育政策，由有关部门批准执行。在批准休假期间内，工资不得低于７５％，工龄连续计算。
　　女职工较多的单位，应按国家有关规定，以自办或联办的形式，逐步建立有专人管理的托儿所、幼儿园。

　　**第十二条**　对女职工较多的单位在新建、改建、扩建工程中，要按照《工业企业设计的卫生标准》的规定，将孕妇的休息室、婴儿哺乳室、女工卫生室（冲洗室）等女职工劳动保护设施列入基建规划，做到同时设计、同时施工、同时投入生产和使用。
　　要重视一线女职工的厕所建设，应为蹲式；女职工浴室要符合卫生条件，采取淋浴式。

　　**第十三条**　对女职工每一至两年进行一次妇科疾病及乳腺疾病的普查，建立健康档案，对患者积极给予治疗。经合同医院证明，患更年期综合症的女职工，不适合现岗位工作的，可以照顾安排适当工作。

　　**第十四条**　女职工劳动保护的权益受到侵害时，有权向所在单位的主管部门或者当地劳动部门提出申诉，受理申诉的部门应当自收到申诉书之日起３０日内作出处理决定。属于劳动争议的，依照《[劳动法](https://www.pkulaw.com/chl/15fe2d94afee49a2bdfb.html)》规定办理。女职工对处理决定不服的，可以在收到处理决定书之日起，１５日内向人民法院起诉。

　　**第十五条**　对违反本办法侵害女职工劳动保护权益的单位负责人及其直接责任人员，其所在单位的主管部门，应当根据情节轻重，进行批评教育或给予行政处分，并责令该单位给予被侵害女职工合理的经济补偿；构成犯罪的，由司法机关依法追究刑事责任。

　　**第十六条**　各单位对本办法的执行情况，由劳动人事部门检查落实，工会、卫生、劳动保护部门对本办法的执行情况进行监督。

　　**第十七条**　女职工违反国家有关计划生育规定的，其劳动保护应按照国家有关计划生育规定办理，不适用本办法。

　　**第十八条**　本办法由交通部负责解释。

　　**第十九条**　本办法自１９９５年７月１日起实施。

© 北大法宝：（[www.pkulaw.com](https://www.pkulaw.com/)) 专业提供法律信息、法学知识和法律软件领域各类解决方案。北大法宝为您提供丰富的参考资料，正式引用法规条文时请与标准文本核对。
欢迎查看所有[产品和服务](https://www.pkulaw.com/helps/2.html)。法宝快讯：[如何快速找到您需要的检索结果？](https://www.pkulaw.com/helps/70.html)[法宝](https://www.pkulaw.com/helps/69.html)V6有何新特色？

扫描二维码阅读原文

**原文链接：**<https://www.pkulaw.com/chl/16c62605e72b677fbdfb.html>
